# Tracheal Rhinosporidiosis: A Report of a Rare Case From Odisha, India

**DOI:** 10.7759/cureus.85997

**Published:** 2025-06-14

**Authors:** Pradipt Sahoo, Chandan Mohapatra, Monalisa Sahu, Khageswar Rout, Arjyama Banerji, Arnav B Kapoor

**Affiliations:** 1 Otolaryngology-Head and Neck Surgery, Kalinga Institute of Medical Sciences, Bhubaneswar, IND; 2 Cardiothoracic Surgery, Kalinga Institute of Medical Sciences, Bhubaneswar, IND; 3 Infectious Diseases, Yashodha Hospitals, Hyderabad, IND; 4 Otolaryngology–Head and Neck Surgery, Kalinga Institute of Medical Sciences, Bhubaneswar, IND

**Keywords:** airway obstruction, cardiopulmonary bypass, mesomycetozoea, rhinosporidium seeberi, tracheal rhinosporidiosis

## Abstract

We report a very rare case of tracheal rhinosporidiosis having multiple sites of involvement in the tracheal lumen, almost completely obstructing the lumen near the carina, which was removed by using peripheral cardiopulmonary bypass (CPB) with a multidisciplinary team approach involving the ENT and cardiothoracic vascular surgery (CTVS) departments.

Since the location of the mass was in the lower trachea and there was a higher risk of bleeding, CPB was done. An open approach was considered for the surgery. A U-shaped neck incision was given, and the trachea was opened at the level of the third, fourth, and fifth tracheal rings. A Hopkins 0° 3 mm 14 cm endoscope was used to visualize the mass, which almost occupied the whole lumen of the trachea near the carina. A rhinosporidiosis mass was found to be attached to the anterolateral aspect of the trachea. The stump of the mass was cauterized by bipolar suction cautery and coblator, and the mass was delivered. The trachea was closed with a cuffed tracheostomy tube (7.5 mm inner diameter (ID)) in situ. After confirming satisfactory ventilation, the patient was weaned off CPB. The duration of CBP was 120 minutes, and the duration of surgery was 80 minutes. The patient was kept in the ICU for 24 hours after surgery. The tracheostomy tube was removed on the fifth post-op day. The patient had an uneventful recovery. The histopathological study of the resected specimen showed sporangia filled with small, round endospores of *Rhinosporidium seeberi*. We report a rare case of rhinosporidiosis that had to be managed in an unconventional method due to the site and size of the pathology, and to minimize the risk to the patient.

## Introduction

Rhinosporidiosis is a granulomatous infection caused by *Rhinosporidium seeberi*, affecting the mucous membrane and skin. It is a eukaryotic aquatic protistan microbe that belongs to the newly described class *Mesomycetozoea *and lies at the animal-fungus boundary [1]. The disease is spread across the tropical regions and is most prevalent in Sri Lanka and Southern India [2]. The first case of tracheobronchial rhinosporidiosis was reported in 1956 by Thomas et al. [3]. The most common sites involved include the mucous membrane of the nose and nasopharynx, and the other less common sites are the eye, oral cavity, oropharynx, uvula, palate, epiglottis, maxillary antrum, ear, scalp, genitals, and skin [4]. Involvement of the trachea and bronchus is extremely rare. Patients with tracheobronchial rhinosporidiosis may present with cough, wheeze, breathlessness, stridor, hemoptysis, or lung collapse. If there is a critical airway compromise, it may rapidly progress to respiratory distress and even death [5, 6]. It poses several challenges in diagnosis and surgical management, as well as in anesthesia delivery during surgery. Other diseases like tuberculosis and neoplastic lesions of the lower respiratory tract should be excluded before confirming the diagnosis of tracheal rhinosporidiosis. Flexible bronchoscopy is performed, and contrast-enhanced CT imaging of the neck and thorax provides details about the location and extent of the lesion, thus helping in accurate diagnosis [7].

Different modes of management of tracheobronchial rhinosporidiosis include flexible and rigid bronchoscopic snaring, argon plasma coagulation, laser, tracheostomy, and surgical excision by co-ablation [8]. Tumors involving more than 80% to 90% of the tracheal lumen and situated just near the carina are difficult to anesthetize and resect. Peripheral cardiopulmonary bypass (CPB) has been considered to provide oxygenation support while performing such surgery [9]. We report a very rare case of tracheal rhinosporidiosis having multiple sites of involvement in the tracheal lumen, almost completely obstructing the lumen near the carina, which was removed by using peripheral CPB, with a multidisciplinary team approach involving the ENT and cardiothoracic vascular surgery (CTVS) departments.

## Case presentation

A 40-year-old male patient presented to the ENT department with respiratory distress for 15 days, along with intermittent hemoptysis and epistaxis for three months. The patient had a history of nasal rhinosporidiosis, for which he was operated on about two years back elsewhere. He had a history of bathing in local ponds prior to the previous surgery.

**Figure 1:**
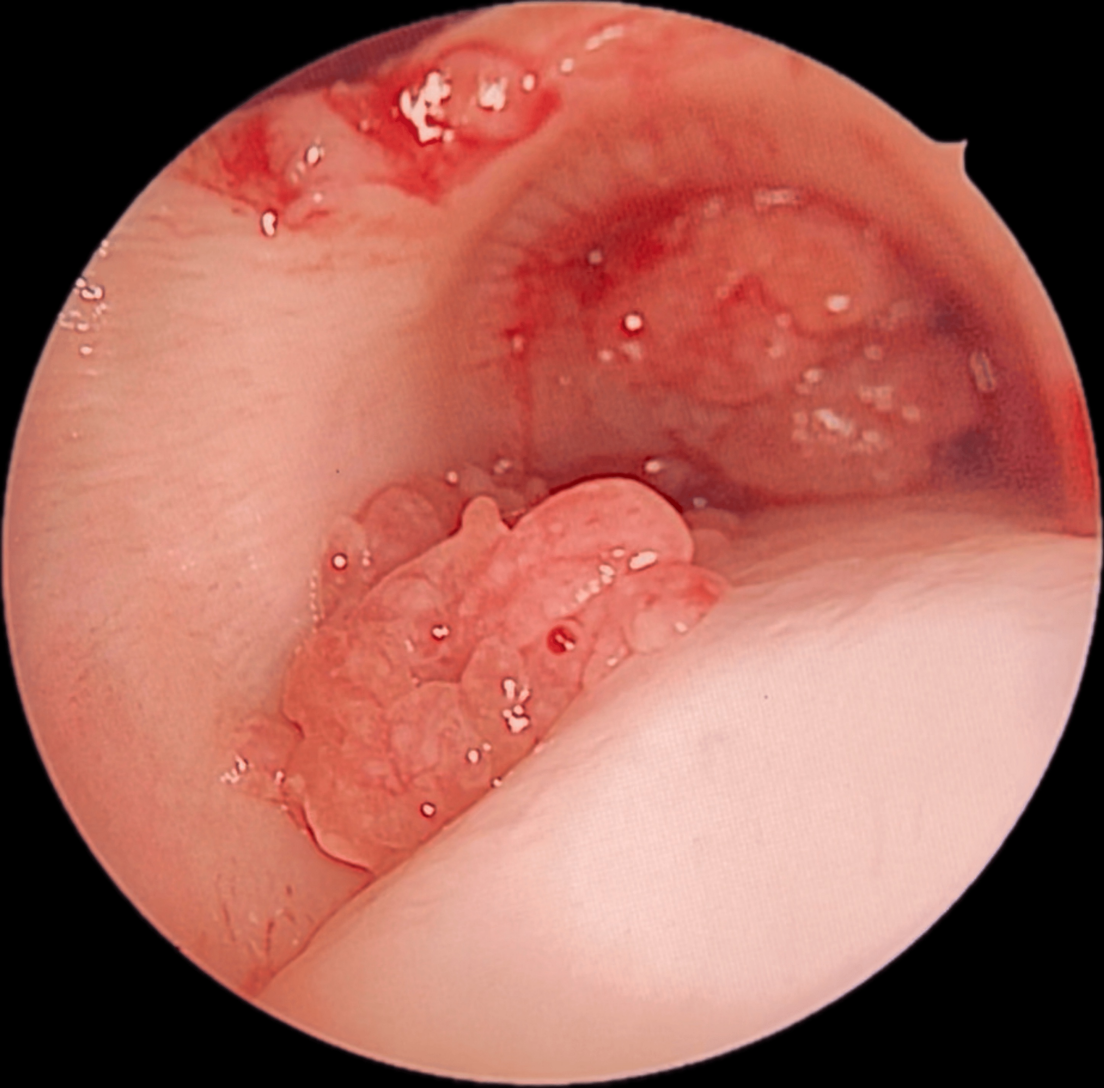
Flexible fiberoptic bronchoscopy showing two red, granular masses in the lower part of the trachea

A nasal endoscopy was performed, which revealed a strawberry-like mass attached to the posterior end of the right inferior turbinate and nasopharynx. Flexible fiberoptic bronchoscopy was done, which showed two red, granular masses in the lower part of the trachea (Figure 1).

**Figure 2:**
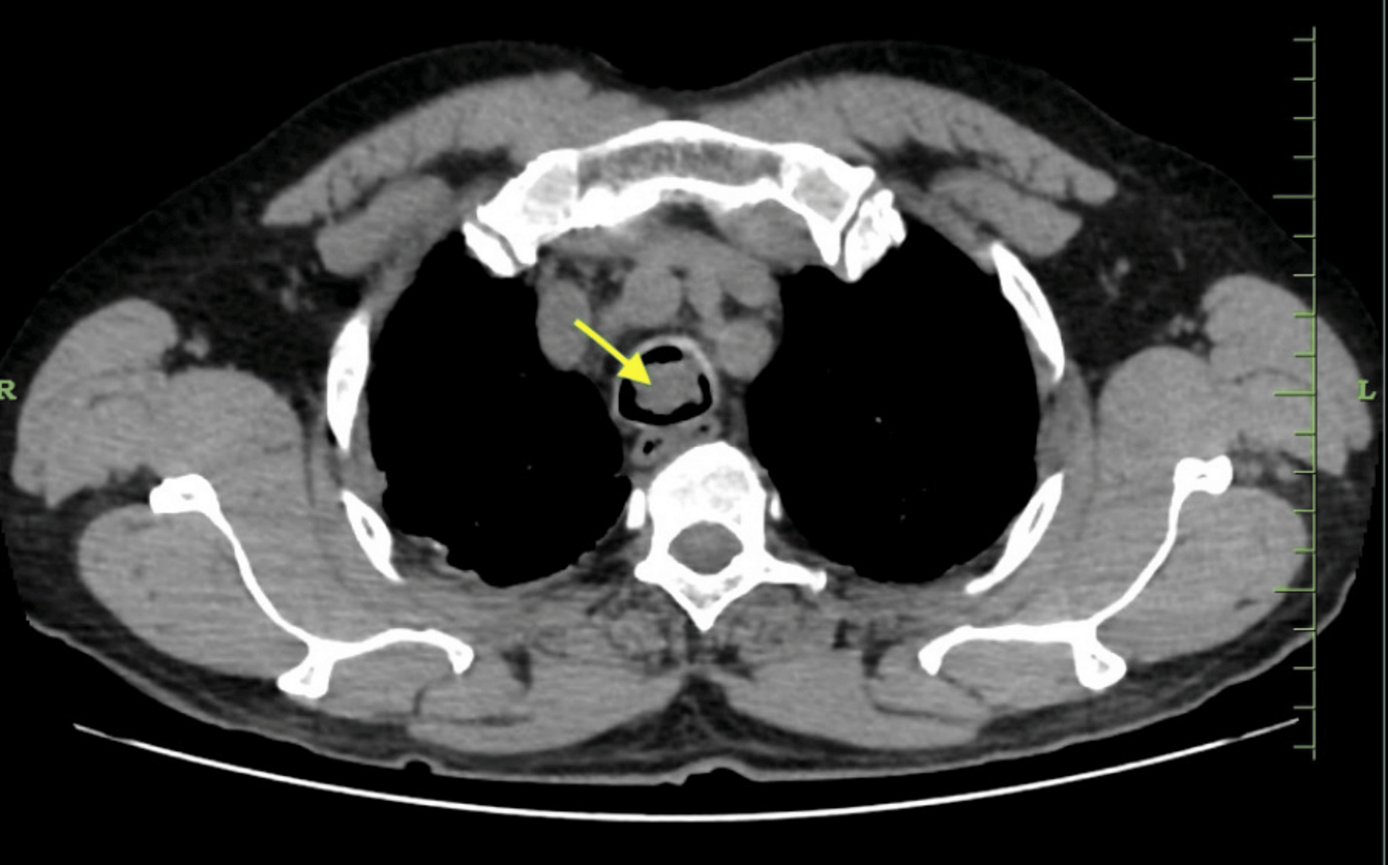
Contrast-enhanced computed tomography (CECT) showing a mass in the trachea (yellow arrow)

The lowermost mass, which was nearer to the carina, almost completely occluded the tracheal lumen. Contrast-enhanced computerized tomography (CECT) of the neck and thorax showed soft tissue opacification in the lower trachea extending up to the carina (Figure 2) with a dimension of 14 mm x 11 mm x 9 mm. Since the location of the mass was in the lower trachea, with a higher risk of bleeding, tracheostomy and general anesthesia by endotracheal intubation were deferred.

Hence, for the management of this difficult case, a multidisciplinary team including ENT, CTVS, and cardiac anesthesiology was formed. CPB was done to overcome these difficulties of intubation.

The nasopharyngeal component of rhinosporidiosis was removed with a co-ablation (EVAC 70 Xtra, Smith & Nephew, London, UK) wand through the oropharynx after application of a Boyle-Davis mouth gag in the supine position and transnasal visualization via a 4 mm 0° endoscope, followed by cauterization of the attachment points.

**Figure 3:**
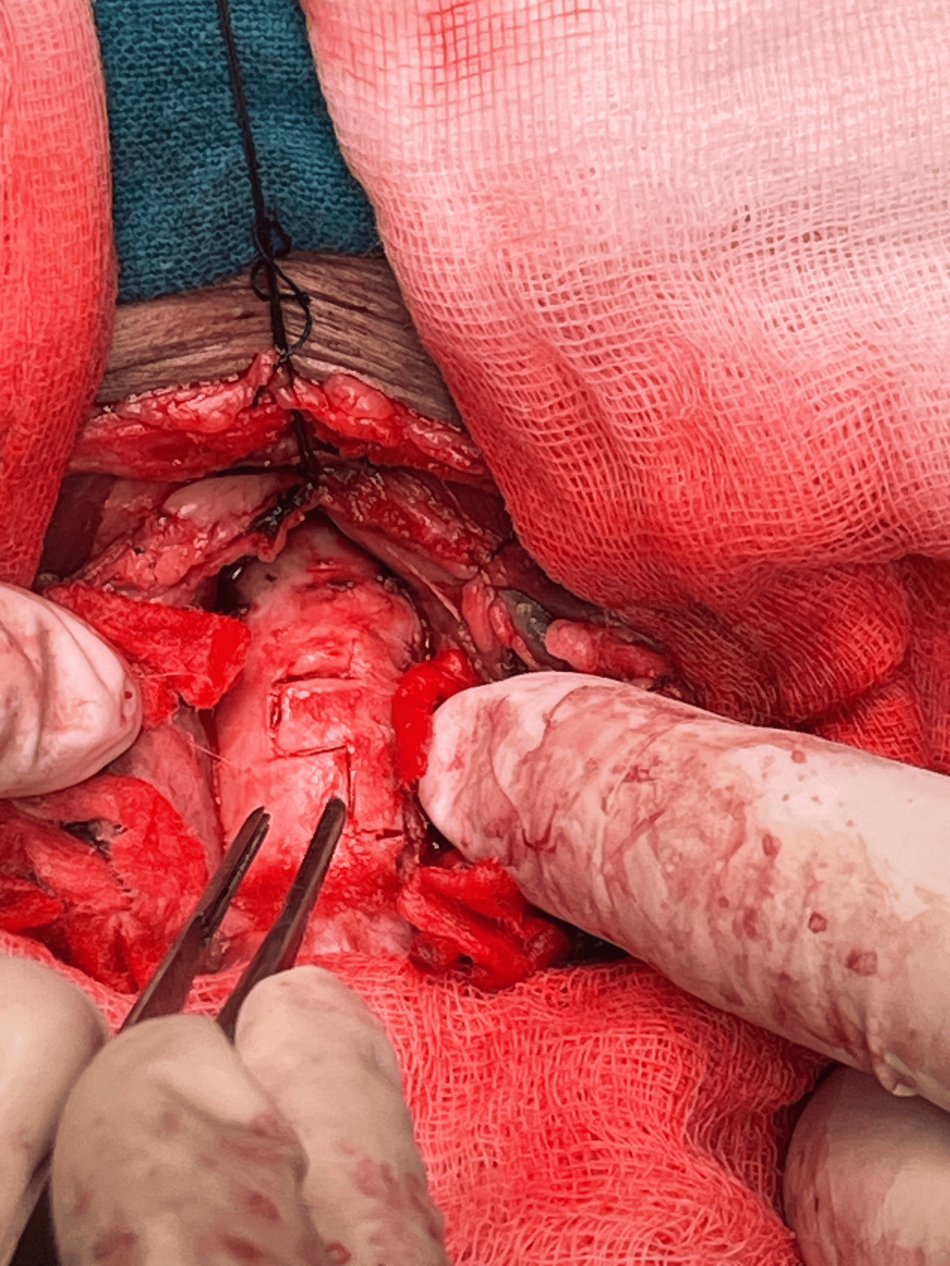
Tracheal incision

A rigid bronchoscope was not used due to the large size of the mass, difficulty in controlling bleeding, and inadequate instrumentation in the limited space available. Hence, an open approach was considered for the surgery. A U-shaped neck incision was given, and the trachea was opened at the level of the third, fourth, and fifth tracheal rings (Figure 3). 

**Figure 4:**
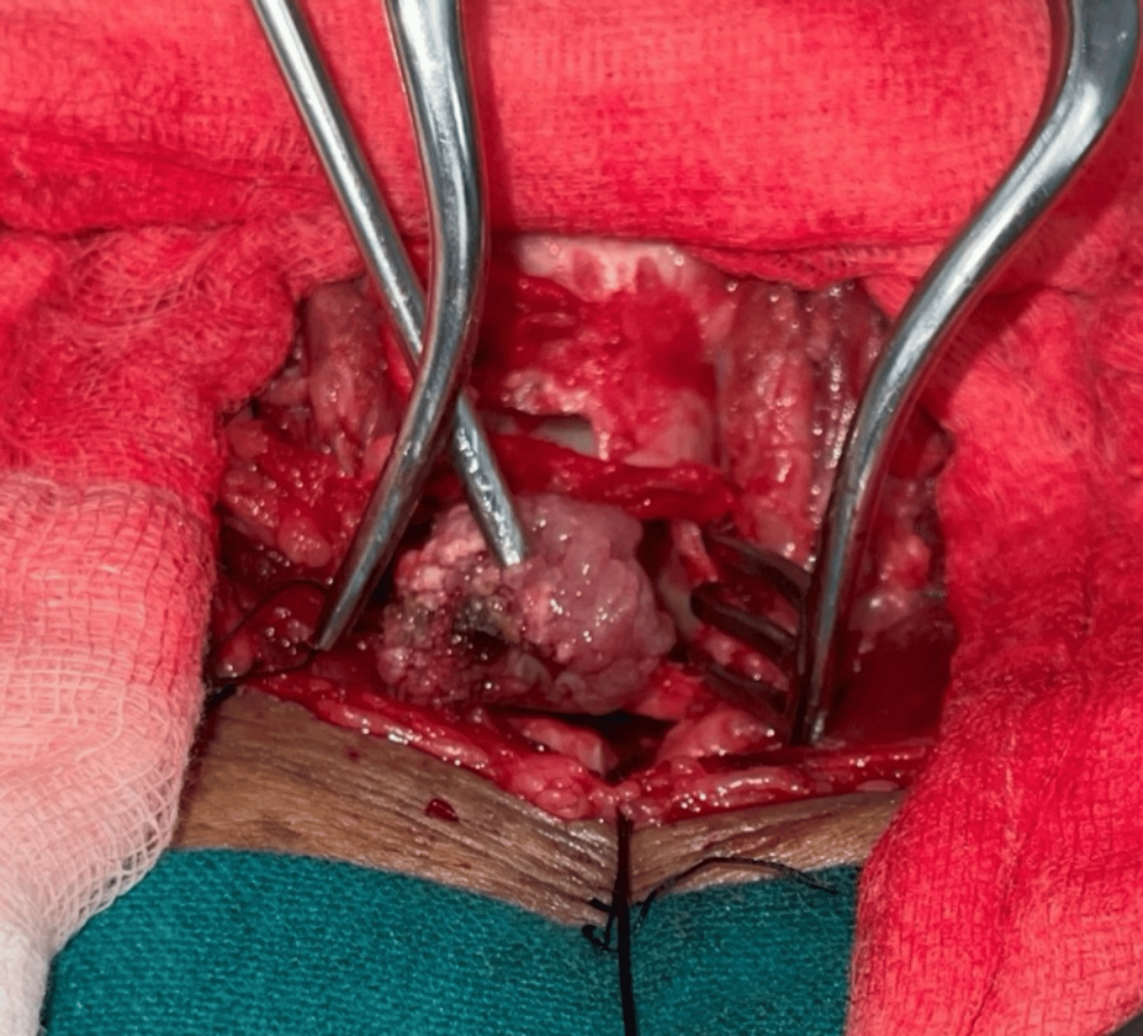
Delivery of the mass

A Hopkins 0 °3 mm 14 cm endoscope was used to visualize the mass, which almost occupied the whole lumen of the trachea near the carina. The rhinosporidiosis mass was found to be attached to the anterolateral aspect of the trachea. The stump of the mass was cauterized by bipolar suction cautery and co-ablator (EVAC 70 Xtra HP wand, Smith & Nephew), and the mass was delivered (Figure 4).

**Figure 5:**
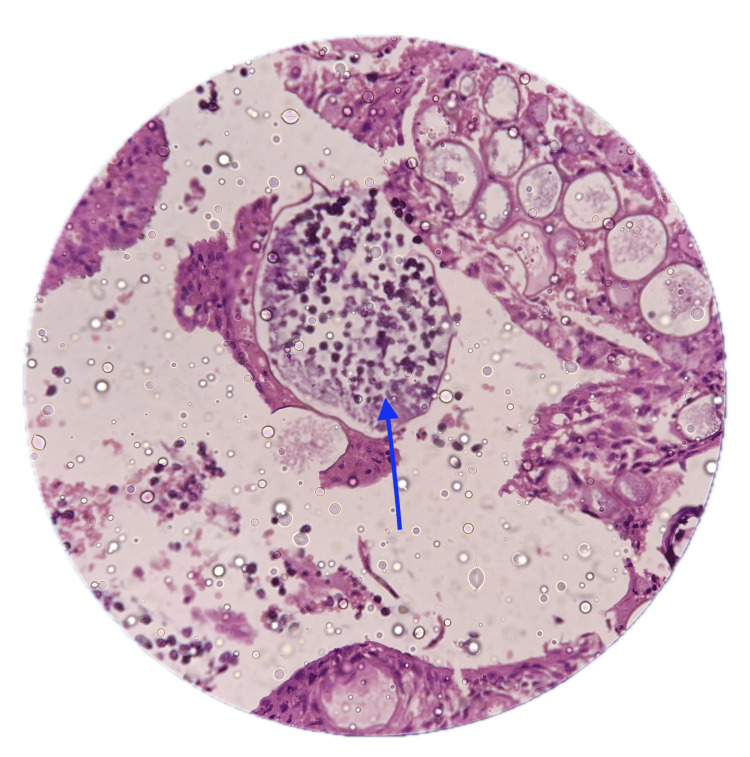
Histopathology slide showing sporangia filled with small round endospores of Rhinosporidium seeberi (blue arrow)

The trachea was closed with a cuffed tracheostomy tube (7.5 mm inner diameter (ID)) in situ. After confirming satisfactory ventilation, the patient was weaned off CPB. The duration of CPB was 120 minutes, and the duration of surgery was 80 minutes. The patient was kept in the ICU for 24 hours after surgery. The tracheostomy tube was removed on the fifth post-op day. The patient had an uneventful recovery. The histopathological study of the resected specimen showed sporangia filled with small round endospores of *Rhinosporidium seeberi* (Figure 5).

The patient was followed up one week, one month, and three months from the date of discharge, and no lesions were visualized in the nose, nasopharynx, trachea, as well as the bronchi. 

## Discussion

Rhinosporidiosis is more common in men in the middle age group [10]. The possible modes of infection are direct contact with spores via dust, swimming in stagnant water contaminated with spores, or infected clothing [11]. A breach in the mucosa is essential for rhinosporidiosis to become established [5]. Dislodgement of the rhinosporidiosis mass from the upper respiratory tract during the process of removal and implantation at the new site in the lower respiratory tract is the usual mechanism of tracheobronchial rhinosporidiosis. Involvement of the trachea, in this case, could be due to the implantation of spores from the nose and nasopharynx during the previous surgery. CECT of the neck and thorax was done to know the lower extension of the mass as well as its vascularity. The most effective treatment for rhinosporidiosis is complete surgical excision. Wide tracheostomy, tracheal retractor, and use of a 0° 3 mm endoscope give good access for a three-handed technique to control bleeding and rhinosporidiosis mass removal.

In our case, the rhinosporidiosis mass was large, having multiple attachment sites, and the tracheal lumen was almost completely occupied by it, thus warranting a longer duration of surgery. It was also associated with a higher risk of excessive bleeding, which could have led to incomplete removal and implantation of the surrounding mucosa and bronchus. Hence, in our case, general anesthesia by endotracheal intubation was deferred, and heart-lung bypass was used to deliver oxygen to the lungs during the surgery. Open thoracotomy was not considered the preferred approach in our case, as the lower extent of the mass was above the carina, and avoiding the risk of implantation of the spores to surrounding tissues was essential.

As a general consideration, for previously operated cases of rhinosporidiosis presenting with hemoptysis or upper airway obstruction, bronchoscopic evaluation of the upper airways should be done to rule out tracheal and bronchial spread of the disease. Such cases also warrant consideration by an anesthetist, and at least a backup of heart-lung bypass should be present in the center where such a case is planned for surgery. Early diagnosis and identification of lesions and prompt referral to equipped centers are key in such types of cases.

Prevention of recurrence is key in rhinosporidiosis surgery. The instruments used for surgery should not touch the non-affected areas, and mucosal damage should be as minimal as possible. Regular follow-ups are key to ruling out recurrence, with strict emphasis on prevention of pond bathing, especially in rural and endemic areas.

## Conclusions

The use of an endoscope and co-ablation through a wide tracheostomy incision and oxygen delivery by heart-lung bypass gives the surgeon adequate time for proper control of bleeding and complete removal of the mass, thus reducing the chances of postoperative recurrence of rhinosporidiosis. Control of bleeding and debulking of mass by the use of a co-ablator and laser gives an extra advantage to the surgeon in being more convenient during surgery, ensuring complete removal of the mass, and thus minimizing the chances of recurrence.
